# An efficient recyclable magnetic material for the selective removal of organic pollutants

**DOI:** 10.3762/bjnano.7.136

**Published:** 2016-10-13

**Authors:** Clément Monteil, Nathalie Bar, Agnès Bee, Didier Villemin

**Affiliations:** 1 UMR CNRS 6507 LCMT, Normandie Université -ENSICAEN, 6 boulevard du Maréchal Juin, 14050 Caen, France; Tel: +33 2 31 45 28 40; 2 UMR CNRS 8234 PHENIX, Université Pierre et Marie Curie, 75252 Paris Cedex 05, France

**Keywords:** adsorption, magnetic nanoparticles, organic pollutants, phosphonated polyethylenimine

## Abstract

Wastewater cleaning strategies based on the adsorption of materials are being increasingly considered, but the wide variety of organic pollutants at low concentrations still makes their removal a challenge. The hybrid material proposed here consists of a zwitterionic polyethylenimine polymer coating a magnetic core. Polyethylenimine is phosphonated at different percentages by a one-step process and used to coat maghemite nanoparticles. It selectively extracts high amounts of cationic and anionic contaminants over a wide range of pH values, depending on the adjustable number of phosphonate groups introduced on the polymer. After recovering the nanoparticles with a magnet, pollutants are quantitatively released by repeated washing with low amounts of pH-adjusted water. The material can be reused many times without noticeable loss of efficiency and is designed to resist high temperatures, oxidation and harsh conditions.

## Introduction

During the last decades, an increased emphasis was placed on the issue of diffuse contamination of water. Toxic metals and organic pollutants are significant sources of hazard for human health, even at low concentrations [1–3]. Many technologies such as photodegradation, biodegradation, the Fenton process, or extraction by liquid membranes have been developed to eliminate these compounds in wastewater [4–6]. Among them, adsorption-based methods are extensively studied [7–8].

At the same time, the emergence of nanotechnologies has led to a new generation of organic/inorganic nanocomposites with embedded magnetic nanoparticles (NPs) [9–13]. The application of a simple magnetic field is sufficient to collect them, which fosters the development of low-cost recyclable processes. However, while many systems can efficiently remove hazardous metallic ions from waters, the elimination of organic micropollutants is still an issue [11,14]. Most of the materials are exclusively efficient for cationic or anionic molecules, and only few of them have been successfully tested on both [15]. In addition, they often adsorb a limited amount of contaminant, considerably lower than the capacity of activated charcoal.

We report herein a new adsorption process for water remediation, based on partially phosphonated polyethylenimine (PEIP)-coated magnetic nanoparticles (NP-PEIP). The special feature of the PEIP is the presence of numerous ammonium and, more original, phosphonate groups spread on the polymer. Its zwitterionic structure allows the adsorption of any kind of charged contaminant. Unlike many others sorbents, this nanomaterial strongly resists the degradation caused by hydrolysis or oxidation, due to strong covalent Fe–O–P bonds [16]. As a result it can be indifferently used in acidic or basic media, in contrast to other sorbents based on silica shells or coatings with oligosaccharides [17].

## Experimental

### Material and apparatus

Polyethylenimine (25000 Da) was purchased from BASF, phosphorous acid, formaldehyde, hydrochloric acid and sodium hydroxide were obtained from VWR and dyes (methylene blue, MB, and methyl orange, MO) from Sigma-Aldrich. Dialysis tubings were bought from Roth. Absorbance was measured by using a UV–visible Perkin-Elmer Analyst 100 spectrophotometer. The microwave used was a Prolabo Synthewave. A Varian SpectrAA 55 AAS was used to detect potential traces of iron in the supernatants after collecting nanoparticles. Zeta potential measurements were determined by DLS analysis using a Malvern Zetasizer nanoZS model.

### Preparation of the phosphonated polyethylenimine–maghemite material

#### Synthesis of nanoparticles

Maghemite ionic ferrofluid ([Fe] = 10^−2^ mol/L) was prepared by wet alkaline coprecipitation according to the Massart protocol [18–19]. Iron(III) chloride and iron(II) chloride were co-precipitated at a molar ratio of 1:2, in the presence of ammonium hydroxide solution (28%), at room temperature and under mechanical stirring. Then, a solution of iron(III) nitrate in concentrated nitric acid was added at 80 °C under stirring. After the removal of the supernatant, nanoparticles were washed with acetone and diethylether and then redispersed in a controlled volume of water. The pH value of the ionic ferrofluid was about 2, with NO_3_^−^ as counterion. Nanoparticles are spherical, with an average diameter of 7 nm determined by XRD [17].

#### Synthesis of the polyethylenimine phosphonate

Phosphonated polyethylenimine (PEIP) was prepared as previously described [20–21]. An adjustment of the amounts of phosphorous acid and formaldehyde is necessary to obtain phosphonated polyethylenimine with different percentages of phosphonation (P%). We have prepared a large range of phosphonated polyethylenimine with a percentage varying from 5 to 90% [20]. For the calculations, we have considered that a molecule of PEI is constituted of monomers (CH_2_–CH_2_–NH)*_n_* with an average molecular mass of 43. So, for example, to synthesize PEIP with P% = 20, 3.81 g (0.2 equiv, 4.65·10^−2^ mol) of phosphorous acid H_3_PO_3_ were introduced in 10 g of PEI (25000 Da, 4·10^−4^ mol of PEI corresponding to 23.2·10^−2^ mol of monomer CH_2_CH_2_NH) in 30 mL of water, and the mixture was irradiated (150 W) for 1 min in a microwave oven. 10 mL of concentrated HCl and 9.30·10^−2^ mol of a 35% formaldehyde solution were successively added. After 5 min of irradiation, excess of formaldehyde was removed under vacuum and the solution was dialyzed with a nitrocellulose membrane, yielding 85% of PEIP [22].

#### Preparation of NP-PEIP*x* powder

An amount of 5 mL of diluted ferrofluid in water ([Fe] = 10^−4^ mol·L^−1^) were added dropwise to 10 mL of a PEIP solution (30 mg/mL) adjusted to pH 2 with diluted nitric acid under vigorous stirring. After 15 min, sodium hydroxide was added to destabilize the solution. The supernatant was removed and the precipitate redispersed in 10 mL of 1 mol·L^−1^ nitric acid by sonication. Then acetone was added until NP-PEIP precipitated. These were washed successively with acetone and diethylether and then dried in an oven at 120 °C for 24 h.

#### Preparation of stock solutions

MO or MB powders were dissolved in distilled water (5·10^−4^ mol·L^−1^) in order to prepare dye stock solutions.

#### Extraction of methyl orange and methylene blue with NP-PEIP

A particles stock solution was prepared by dispersing 5 mg of NP-PEIP powder in 50 mL of distilled water. All the following experiments (pH value, kinetic, maximum of adsorption…) were repeated five times.

#### pH Adsorption studies

The experiments were carried out for both NP-PEIP20 and NP-PEIP80. A range of samples was prepared by introducing 10 mL of NP-PEIP stock solution in 10 mL of the dye solution. For all of them, the pH was adjusted between 1 and 14 with either diluted nitric acid or sodium hydroxide solution. After 3 h of stirring, particles were collected with a magnet, and the supernatant was removed. The particles were rinsed twice with 1 mL of distilled water, which was afterwards added to the liquid phase. Supernatant absorbance was monitored by UV–visible spectrophotometry. It is noteworthy that OM exhibits acido–basic properties and is used as pH indicator. At pH 14, the OM absorption wavelength does not vary (λ = 465 nm), neither does the molar extinction coefficient ε (Figure S3 in Supporting Information File 1). So, in the case of the MO solution, the supernatants were adjusted to pH 14 by addition of sodium hydroxide before recording the absorption in order to avoid a distortion of the measurements (Figure S4 in Supporting Information File 1). For MB, this problem does not occur and we have established calibration curves according to the pH values (Figure S5 and Figure S6 in Supporting Information File 1).

#### Kinetic studies

The experiments were carried out for both NP-PEIP20 and NP-PEIP80. 15 replicate tests were prepared for each dye, using the same protocol as previously, but with a constant pH value (7 for MO, 14 for MB corresponding to the pH value of maximum adsorption). Evolution of absorbance was monitored by spectrophotometry and recorded at different times, changing the sample between measurements.

#### Washing step

Acidic and basic wash waters were obtained by adjusting distilled water to pH 2 or 14 with diluted nitric acid (1 mol·L^−1^) and a solution of sodium hydroxide respectively (0.1 mol·L^−1^). 10 mL of NP-PEIP stock solution were mixed with contaminant solutions as described above, at the pH value of maximum adsorption (MO: pH 7, MB: pH 14) for 3 h. After that, the particles were re-dispersed in 20 mL of wash water per milligram of NP-PEIP depending on the pollutant: MO was washed with the acidic solution, MB with the basic one. After 5 min of stirring, the particles were collected with a magnet and the supernatant absorbance of each run was monitored by spectrophotometry.

#### Recyclability

Five replicate tests were conducted to guarantee the reproducibility of the results. 20 mg of NP-PEIP (P% = 20) were dispersed in 20 mL of the dye stock solution and vigorously stirred for 4 h. Then, the particles were collected with a magnet and the absorbance of the supernatant was monitored by spectrophotometry. Four washing steps were carried out between each cycle, and the particles were directly reused in the new dye solution batch.

## Results and Discussion

### Magnetic material preparation and conditions of the studies

The novelty of this contribution consists in the use of PEI with phosphonic groups allowing a solid grafting of PEI on the maghemite nanoparticles, by the formation of strong covalent P–O–Fe bonds. The presence of these negative phosphonic groups ensures the stability of NP regardless of the pH value, especially in very basic medium, which is not the case for plain PEI [23].

**Figure 1:**
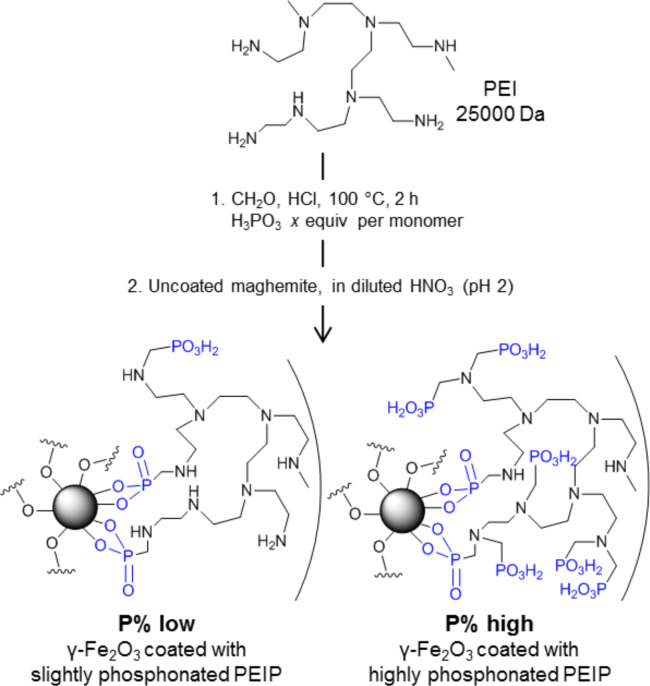
Synthesis of NP-PEIP. The number of phosphonates depends on the number of equivalents of phosphorous acid used.

The phosphonated groups are introduced on PEI through the modification of primary and secondary amines according to the Moedritzer and Irani synthesis [24]. In a previous work we described the synthesis, the coating of maghemite and a characterization of the physicochemical properties of the material [20,22]. As illustrated in Figure 1, PEIP can be customized to contain more or less phosphonated groups, by varying the percentage of amines modified (P%). Between pH 3 and 10, the zeta potential is positive but decreases with the percentage of phosphonation (see Figure S2 in Supporting Information File 1). After the point of zero charge (PZC), the phosphonates induce a negative charge, whereas non-phosphonated PEI-NP display a zeta potential of 0 mV, making PEI-NP unstable at basic pH.

The efficiency of the NP-PEIP in removing pollutants was determined by the adsorption of two organic dyes used as contaminants models: the positively charged (regardless of the pH value) methylene blue (MB) and the negatively charged (from pH 3.4 to basic pH) methyl orange (MO). Such dyes are pollutants themselves. They are widely used for industrial purposes, especially MB in the textile industry [25], they reduce light penetration and photosynthesis in the effluents. Their removal remains a challenge [26].

With the aim of evaluating the interactions of the NP-PEIP material with pollutants in very low quantities, which are considerably harder to remove, the concentration of the dyes was kept below 5·10^−4^ mol·L^−1^ in all the experiments. This choice of low concentration (which is not a strict limitation) is dictated by the need to be close to the most common pollutions that are often diffuse and characterized by low concentration levels. Moreover, the MO and MB calibration curves have been evaluated (data available in Figures S3–S6 in Supporting Information File 1) and show that their absorptions vary significantly either with the pH or with the concentration. It can be observed that working at low concentrations allows one to avoid the di- or trimerization of MB, which dramatically impacts the wavelength of the maximum absorption.

### Determination of the optimal conditions

First, we studied the pH value at which a maximum of dye is loaded on the particles. Adsorptions in solutions of MO or MB were carried out at different pH values using two types of particles as sorbents: one coated with a slightly phosphonated polymer (P% = 20, NP-PEIP20) and another coated with a highly phosphonated polymer (P% = 80, NP-PEIP80).

**Figure 2:**
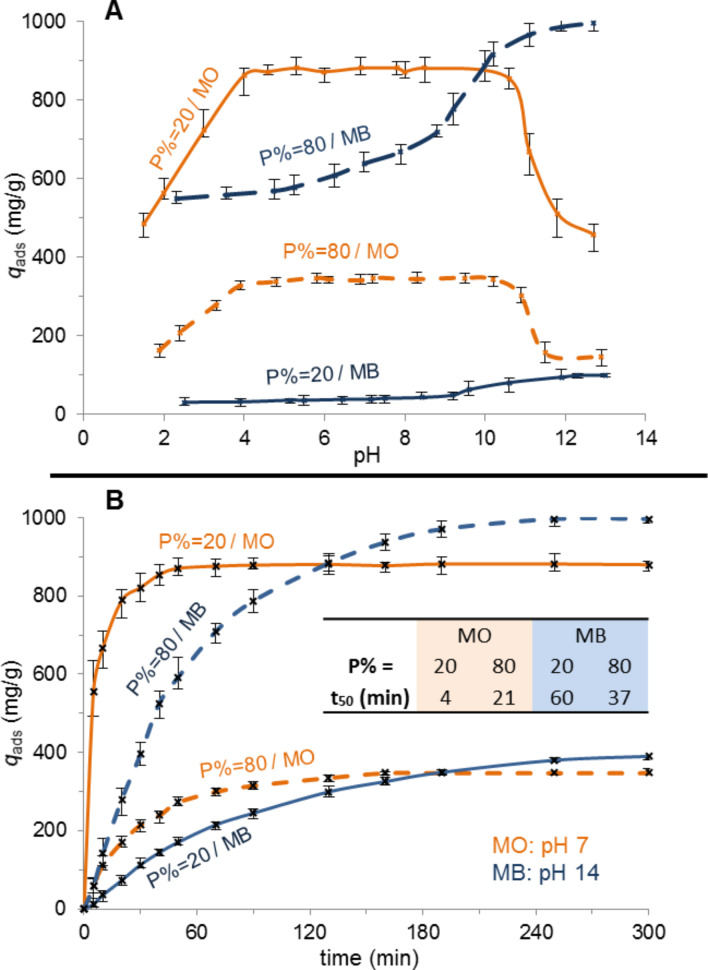
A) pH Values at which NP-PEIP20 and NP-PEIP80 adsorb a maximum amount of dye. MO is better adsorbed in the range of pH from 4 to 10. P% does not impact the pH range but the amount of removed dye. For MB, the higher the P% is, the better the extractions are, regardless of the pH value. Maximum amounts are removed at basic pHs. B) Adsorption of MO is faster than that of MB, regardless of the percentage of phosphonate groups.

Figure 2A shows that the maximum of the anionic dye MO is recovered between pH 4 and 10. The percentage of phosphonation does not significantly affect the pH range of efficiency, only the amount of MO adsorbed: 870 mg/g for NP-PEIP20, whereas 350 mg/g only for NP-PEIP80. On the other hand, the adsorption of the cationic dye MB by NP-PEIP20 is constantly low (≤40 mg/g) below pH 10. We assume that the positive charges on the ammonium groups cause electrostatic repulsions with the cationic dye. In the case of NP-PEIP80, the PEI carried many phosphonate groups and the resulting numerous negative charges decrease the repulsion between ammonium and MB. Consequently, up to 700 mg/g of MB can be extracted by NP-PEIP80. The MB adsorption increases up to 1000 mg/g above pH 10.

The kinetic curves, presented Figure 2B, highlight the fast MO extraction regardless of the P% value. Indeed, 50% of maximum adsorption capacity is reached after only 4 min and 21 min in the two cases. MB is removed more slowly: 37 and 60 min for P% = 80 and P% = 20 respectively, are needed to load half of the pollutants on the NP-PEIP. That confirms the assertion that the adsorption rate is strongly dependent on the amount of phosphonate groups on the polymer. First measurements were monitored only after five minutes: This time was necessary to ensure the collection of all the NP-PEIP with the magnet. It can be explained by the dispersion state of the particles in solution, and the nanometric size. The sensitivity to the magnetic attraction is lower for nanometre-sized beads than for micro- or millimetre-sized beads in which many maghemite nanoparticles are often incorporated in a polymeric network.

### Interpretation of the pH-responsive pollutant extraction

**Figure 3:**
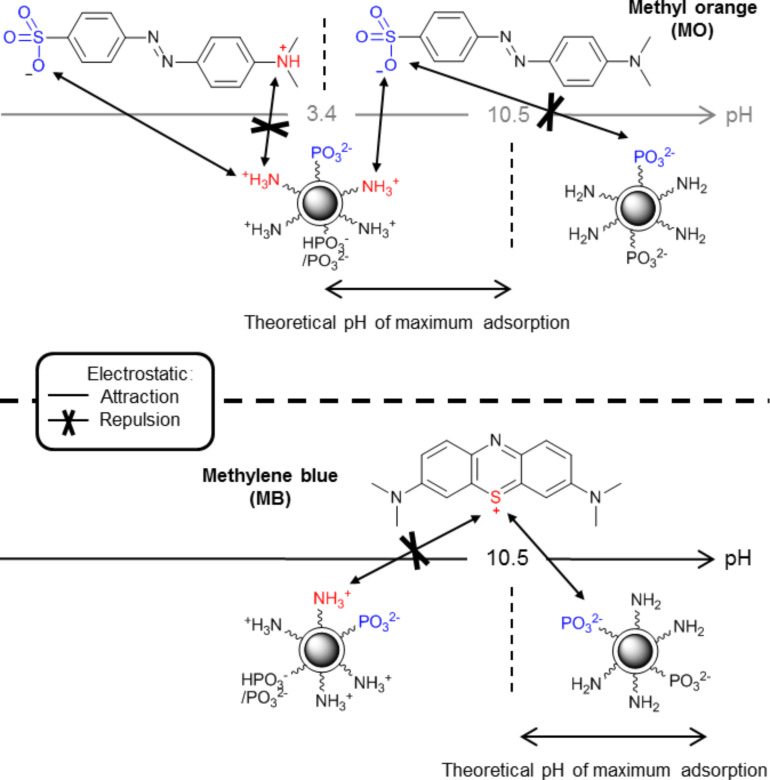
Prediction of the best range of pH values for electrostatic attractions between NP-PEIP and MO and MB dyes. Only the major interaction (i.e., ammonium) is represented. For MO, an interaction occurs with sulphates and ammonium groups, until the p*K*_a_ (NH_3_^+^/NH_2_) is reached. Cationic MB is attracted by the phosphonate groups introduced on PEI at basic pH values.

To understand these results, a diagram predicting the specific interactions between particles and dyes according to the pH value is showed in Figure 3. Phosphonate functions on the NP-PEIP are in the monodeprotonated RPO_3_H^−^ form above pH 2, and undergo a second deprotonation (RPO_3_^2−^) around pH 6.5–7.0. In the presence of both ammonium and phosphonate groups, only ammonium interactions are represented because they are always in the majority, therefore they have strongest influence on the behaviour of the particles.

We can predict that the MO extraction is maximized between pH 3.4 (above its p*K*_a_) and pH 10.5, corresponding to the amine deprotonation on the NP-PEIP. In this range of pH, the electrostatic interactions between numerous ammonium and MO sulfonate groups are at the maximum intensity. Concerning the MB, the sulfur atom is always positively charged. The adsorption therefore increases after the second deprotonation of phosphonate, and is maximized above p*K*_a_ of the ammonium/amine deprotonation, where repulsive forces are negligible. All of these results show that, in the case of maghemite coated with phosphonated PEI adsorbing MO and MB, electrostatic interactions are predominant.

### Quantities of pollutant extracted according to the percentage of phosphonation

**Figure 4:**
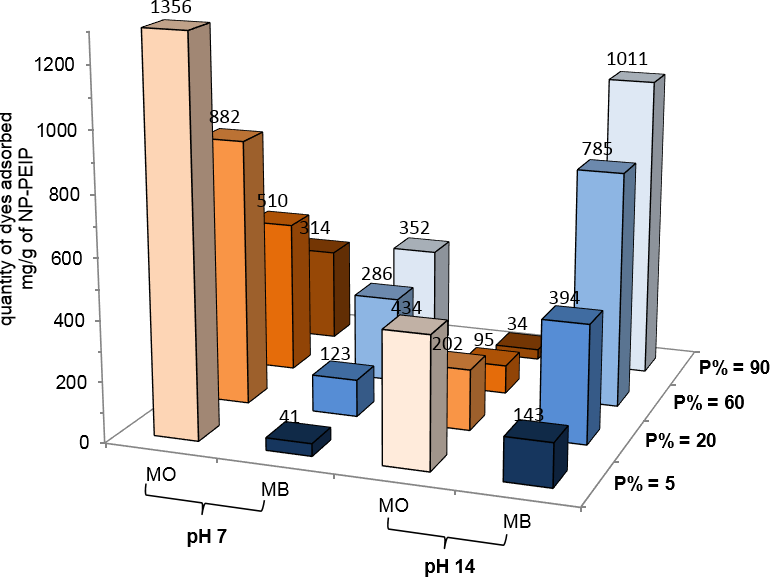
Removed amounts of MO and MB dyes by NP-PEIP*x* at pH 7 and 14. P% significantly affects adsorption of MB and MO. At neutral pH, much more MO is eliminated than MB, while the contrary is observed at basic pH.

Performances of remediation for nanoparticles coated with different polymers (P% = 5, 20, 60, 90) are summarized Figure 4. The maximum efficiency is reached at pH 7 for P% = 5, where the system can remove up to 1356 mg·g^−1^ of MO. When P% increases, the amount of adsorbed dyes, *q*_max_, drop: 882 mg·g^−1^, 510 mg·g^−1^ and up to 314 mg·g^−1^ for NP-PEIP20, NP-PEIP60 and NP-PEIP90, respectively. Referring back to Figure 3, the repulsion between negative charges increases with the number of introduced phosphonate groups. At pH 14, we observe a similar evolution, but all values of *q*_max_ are much lower because of the absence of electrostatic attraction, whereas all the amino groups are deprotonated.

The phosphonation rate also strongly influences the adsorption of MB. The value of *q*_max_ is increased to the seven-fold between P% = 5 and P% = 90 at pH 14 with a maximum of 1011 mg·g^−1^. This amount is higher than the commercial activated charcoal (980 mg·g^−1^) and among the largest measured extracted amounts of BM dye [27]. This high *q*_max_ can be attributed to the composition of NP-PEIP: PEIP represents more than 80% of the particles total weight [20], and under the same conditions maghemite does not adsorb more than 1 mg/mg. In addition, each monomeric unit of the PEIP potentially interacts with charged pollutants through amine or phosphonate groups.

### Washing steps and recyclability of the magnetic particles

**Figure 5:**
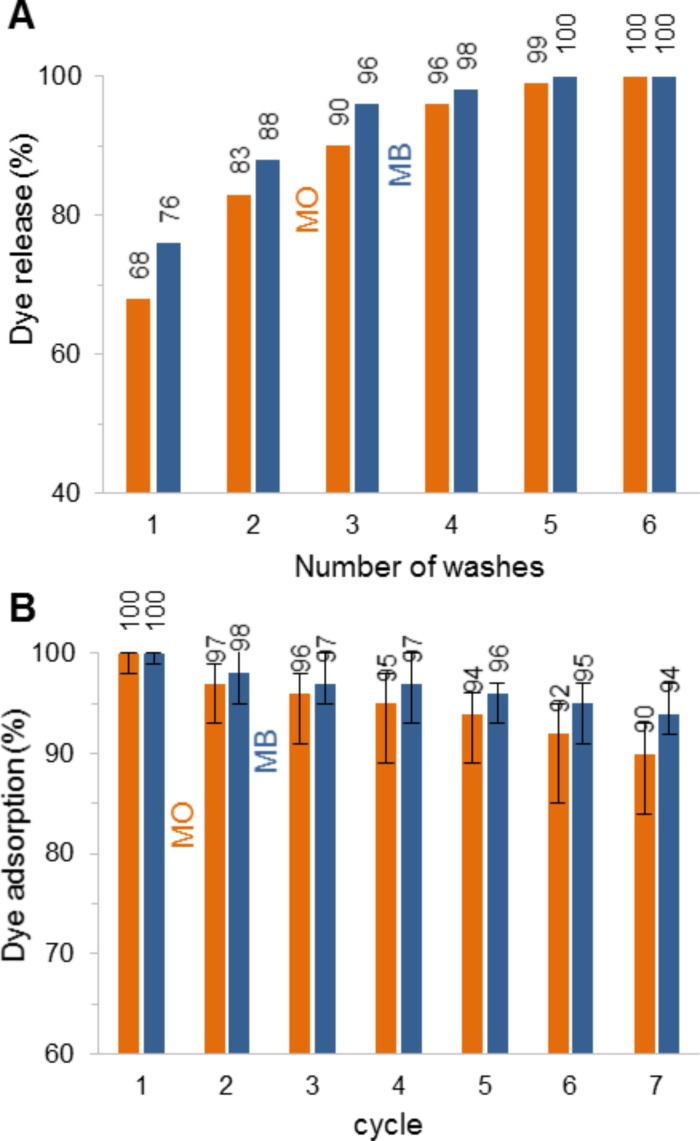
A) Cumulative quantities of dyes released after each wash. MO is desorbed from NP-PEIP05 with sodium hydroxide aqueous solution, MB from NP-PEIP90 with diluted nitric acid. B) The efficiency of the dyes extraction remains high after several adsorption/desorption cycles.

The data collected in Figure 4 reveal that the adsorption of MO with NP-PEIP05 is reduced to one third between pH 7 and 14. On the opposite, the MB adsorption falls from 1011 mg·g^−1^ to 352 mg·g^−1^ with NP-PEIP90 when the pH value decreases from 14 to 7. Consequently, the washing process is easy to implement, by successive redispersions of collected particles in basic or acidic water, for anionic MO and cationic MB pollutants, respectively. The washing solutions consist of water adjusted to pH 7 or 14 with nitric acid or sodium hydroxide. Moreover, only 20 mL are enough to wash up to 1 g of magnetic particles. Figure 5A shows that after a full adsorption of MO by NP-PEIP05 (orange bars), more than 68% of dye are released after the first wash and 83% after two successive washes. NP-PEIP90 loaded with MB (blue bars) release 76% and 88% after the first and the second wash, respectively. Consequently, we can reasonably estimate that four washes are enough to obtain a reusable material practically purified.

NP-PEIP05 and NP-PEIP90 were also evaluated in several adsorption/desorption cycles (Figure 5B). A little loss of activity for MO and MB was observed but more than 90% of contaminants were removed after the seventh cycle. The loss in magnetic particles between cycles is negligible compared to the amount of pollutant extracted and in addition, no traces of iron were detected by atomic absorption spectroscopy in the supernatant. The decreasing of effectiveness is already under study.

## Conclusion

The modulation of positive and negative charges of phosphonated polyethylenimine, through the controlled insertion of phosphonate groups on polyethylenimine, can be considered as an advantage for adsorption of organic pollutants. By adjusting the percentage of phosphonation, the pH value and the decontamination time, performances similar to active charcoal are obtained, but with a highly selective adsorption of cationic and anionic contaminants. This recyclable material obtained by an easy and reproducible single-step process is particularly designed for water treatment. Moreover, the PEIP polymer is perfectly adaptable for a future use in other systems such as microbeads or membranes.

## Supporting Information

Supporting information includes a TEM image of the synthetized maghemite nanoparticles, evolution of the zeta potential of maghemite, PEI nanoparticles and PEIP-coated nanoparticles as a function of the pH value, and evolution of the maximum absorption wavelengths and molar extinction coefficients of methyl orange and methylene blue, and corresponding calibration curves.

File 1 Additional experimental data.

## References

[1] Cotruvo J A (1985). Sci Total Environ.

[2] Richardson S D, Ternes T A (2011). Anal Chem.

[3] Schwarzenbach R P, Escher B I, Fenner K, Hofstetter T B, Johnson C A, von Gunten U, Wehrli B (2006). Science.

[4] Martínez-Huitle C A, Brillas E (2009). Appl Catal, B: Environ.

[5] Rivas B L, Pereira E D, Palencia M, Sánchez J (2011). Prog Polym Sci.

[6] Crini G (2006). Bioresour Technol.

[7] Liu P, Zhang L (2007). Sep Purif Technol.

[8] Dias J M, Alvim-Ferraz M C M, Almeida M F, Rivera-Utrilla J, Sánchez-Polo M (2007). J Environ Manage.

[9] Khin M M, Nair A S, Babu V J, Murugan R, Ramakrishna S (2012). Energy Environ Sci.

[10] Zhong L-S, Hu J-S, Liang H-P, Cao A-M, Song W-G, Wan L-J (2006). Adv Mater.

[11] Koehler F M, Rossier M, Waelle M, Athanassiou E K, Limbach L K, Grass R N, Günther D, Stark W J (2009). Chem Commun.

[12] Obeid L, Bée A, Talbot D, Ben Jaafar S, Dupuis V, Abramson S, Cabuil V, Welschbillig M (2013). J Colloid Interface Sci.

[13] Xu P, Zeng G M, Huang D L, Feng C L, Hu S, Zhao M H, Lai C, Wei Z, Huang C, Xie G X (2012). Sci Total Environ.

[14] Wang H, Chen Q-W, Chen J, Yu B-X, Hu X-Y (2011). Nanoscale.

[15] Luo X, Zhang L (2009). J Hazard Mater.

[16] Mutin P H, Guerrero G, Vioux A (2005). J Mater Chem.

[17] Zhao H, Xu J, Lan W, Wang T, Luo G (2013). Chem Eng J.

[18] Massart R (1981). IEEE Trans Magn.

[19] Lefebure S, Dubois E, Cabuil V, Neveu S, Massart R (1998). J Mater Res.

[20] Monteil C, Bar N, Moreau B, Retoux R, Bee A, Talbot D, Villemin D (2014). Part Part Syst Charact.

[21] Villemin D, Moreau B, Elbilali A, Didi M-A, Kaid M, Jaffrès P-A (2010). Phosphorus, Sulfur Silicon Relat Elem.

[22] Monteil C, Bar N, Retoux R, Henry J, Bernay B, Villemin D (2014). Sens Actuators, B.

[23] Xu Y, Qin Y, Palchoudhury S, Bao Y (2011). Langmuir.

[24] Moedritzer K, Irani R R (1966). J Org Chem.

[25] Bennani Karim A, Mounir B, Hachkar M, Bakasse M, Yaacoubi A (2010). Rev Sci Eau.

[26] Ali H (2010). Water, Air, Soil Pollut.

[27] Rafatullah M, Sulaiman O, Hashim R, Ahmad A (2010). J Hazard Mater.

