# Supplementary material for: An efficient recyclable magnetic material for the selective removal of organic pollutants
Source: Beilstein J Nanotechnol. 2016 Oct 13;7:1447–53. doi: 10.3762/bjnano.7.136 (PMC5082486; doi:10.3762/bjnano.7.136)
Supplement: File 1 — Additional experimental data. [file Beilstein_J_Nanotechnol-07-1447-s001.pdf]

Supporting Information

for

An efficient recyclable magnetic material for the selective removal of organic pollutants

Clément Monteil[1], Nathalie Bar[1], Agnès Bee[2] and Didier Villemin*[1]

Address: [1]UMR CNRS 6507 LCMT, Normandie Université -ENSICAEN, 6 boulevard du Maréchal Juin, 14050 Caen, France; Tel: +33 2 31 45 28 40 and [2]UMR CNRS 8234 PHENIX, Université Pierre et Marie Curie, 75252 Paris Cedex 05, France

Email: Didier Villemin* - didier.villemin@ensicaen.fr

*Corresponding author

Additional experimental data

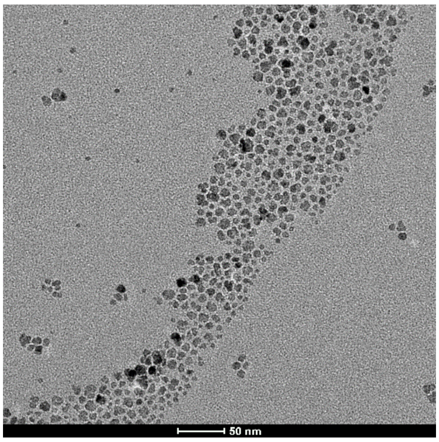

Figure S1: TEM image of the synthetized maghemite nanoparticles.

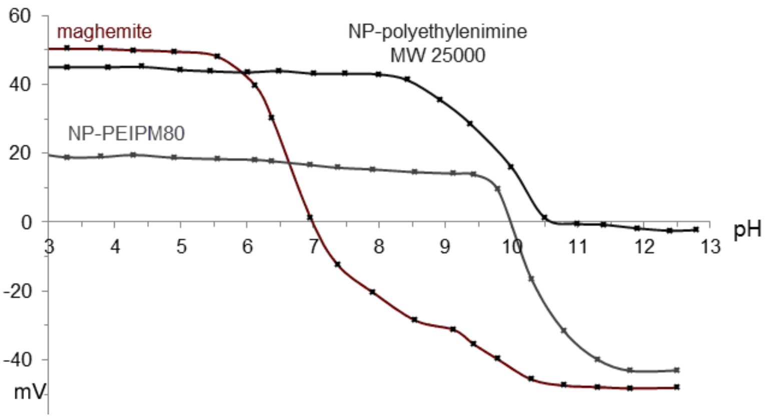

Figure S2: Evolution of the zeta potential of PEIP-coated nanoparticles as a function of the pH value.

Methyl orange (MO)

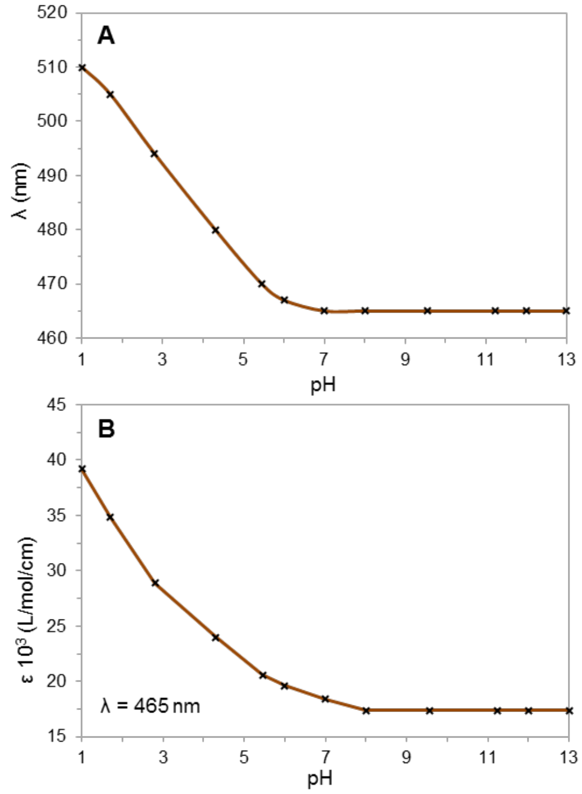

Figure S3: A) Evolution of the maximum absorption wavelength λ_{max} as a function of the pH value. B) Evolution of the molar extinction coefficient at 465 nm as a function of the pH value. To prevent measurement inaccuracy, solutions will be adjusted to pH 14.

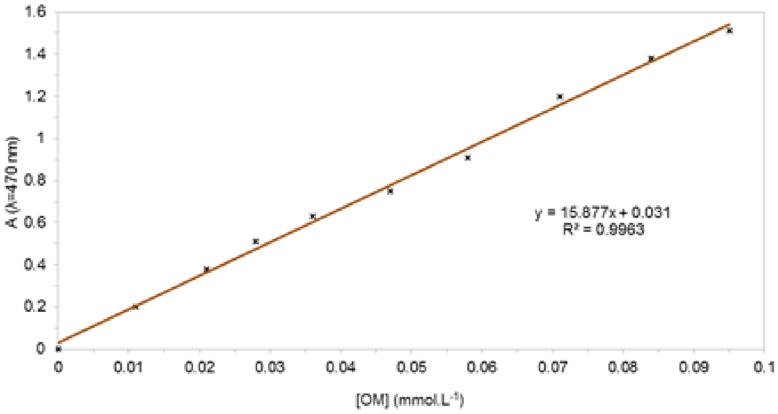

Figure S4: MO calibration curve, at 465 nm, $A = f$([OM]).

Methylene blue (MB)

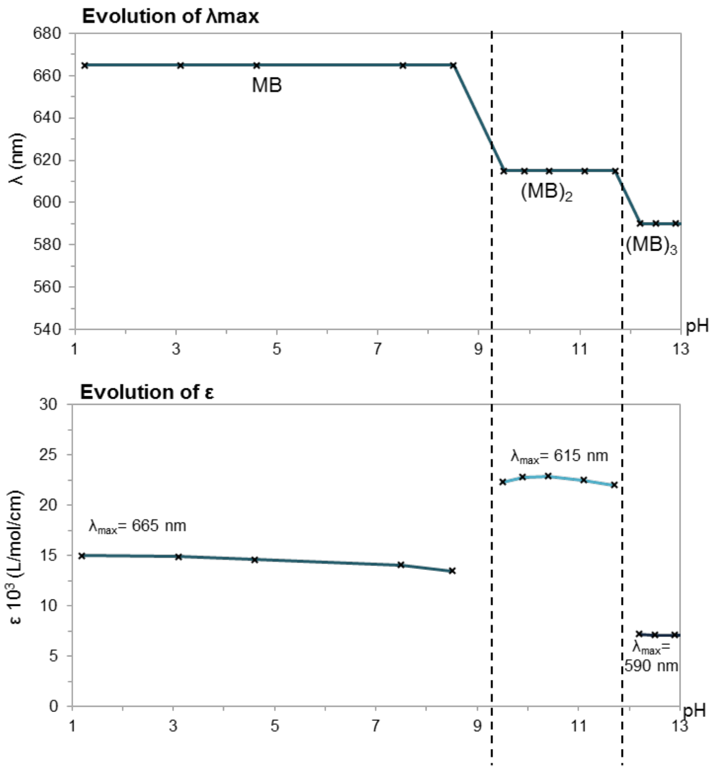

Figure S5: Evolution of the maximum wavelength and molar extinction coefficient as a function of the pH value. We can observe three well-separated domains with different λ_{max} [1].

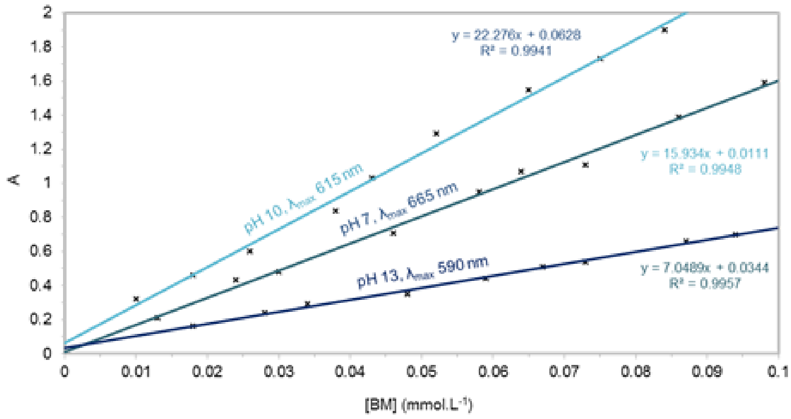

Figure S6: MB calibration curves for the three domains of adsorption (see Figure S5) at pH 7, 10 and 13.

References

[1] Ghosh, A. K. *Curr. Sci.* **1974,** *43,* 655.
